# Randomized Controlled Trial of a Mobile Phone Intervention for Improving Adherence to Naltrexone for Alcohol Use Disorders

**DOI:** 10.1371/journal.pone.0124613

**Published:** 2015-04-24

**Authors:** Susan A. Stoner, Pamela B. Arenella, Christian S. Hendershot

**Affiliations:** 1 Alcohol and Drug Abuse Institute, University of Washington, Seattle, Washington, United States of America; 2 Department of Psychiatry, University of New Mexico, Albuquerque, New Mexico, United States of America; 3 Campbell Family Mental Health Research Institute, Centre for Addiction and Mental Health, Toronto, Ontario, Canada; 4 Department of Psychiatry, University of Toronto, Toronto, Ontario, Canada; 5 Department of Psychology, University of Toronto, Toronto, Ontario, Canada; 6 The Mind Research Network and Lovelace Biomedical and Environmental Research Institute, Albuquerque, New Mexico, United States of America; Asociacion Civil Impacta Salud y Educacion, PERU

## Abstract

**Background:**

Naltrexone is a front-line treatment for alcohol use disorders, but its efficacy is limited by poor medication adherence. This randomized controlled trial evaluated whether a mobile health intervention could improve naltrexone adherence.

**Methods:**

Treatment-seeking participants with an alcohol use disorder (*N* = 76) were randomized to intervention and control conditions. All participants received naltrexone (50 mg/day) with a medication event monitoring system (MEMS) and a prepaid smartphone, and received a daily text message querying medication side effects, alcohol use, and craving. Those in the intervention arm received additional medication reminders and adherence assessment via text message.

**Results:**

The primary outcome, proportion of participants with adequate adherence (defined as ≥80% of prescribed doses taken through Week 8), did not differ between groups in intent-to-treat analyses (*p* = .34). Mean adherence at study midpoint (Week 4) was 83% in the intervention condition and 77% in the control condition (*p* = .35). Survival analysis found that the intervention group sustained adequate adherence significantly longer (*M* = 19 days [95% CI = 0.0–44.0]) than those in the control group (*M* = 3 days [95% CI = 0.0–8.1]) during the first month of treatment (*p* = .04). Medication adherence did not predict drinking outcomes.

**Conclusions:**

These results suggest that in the context of daily monitoring and assessment via cell phone, additional text message reminders do not further improve medication adherence. Although this initial trial does not provide support for the efficacy of text messaging to improve adherence to pharmacotherapy for alcohol use disorders, additional trials with larger samples and alternate designs are warranted.

**Trial Registration:**

ClinicalTrials.gov: NCT01349985

## Introduction

Medication nonadherence is a significant barrier to the pharmacological treatment of alcohol use disorders [1–3]. Naltrexone, one of few approved medications for the treatment of alcohol dependence, delays relapse to heavy drinking, reduces number of heavy drinking days, and increases number of days abstinent [4–6]. Naltrexone is not highly utilized, however, in part due to concerns about non-adherence [7]. It is well recognized that the efficacy of naltrexone is contingent on high adherence [8–11]. For example, effects of naltrexone versus placebo may only be evident only among patients with high adherence [8,11,12], and treatment outcomes correlate with adherence rates [13–16]. Adequate naltrexone adherence has been defined as taking at least 80% of prescribed doses [12, 13, 16]. Naltrexone adherence is generally suboptimal over 12–16 weeks [10,11,13–15,17,18] and worse over longer periods [14]. Marginal adherence compromises treatment effectiveness in real-world settings and the ability to detect medication effects in randomized trials [1,5,8,19].

Randomized trials of naltrexone have included medication monitoring and behavioral interventions to support adherence [5,15,21] but these interventions have rarely been evaluated experimentally [2]. Although behavioral interventions [10,22,23] appear to be beneficial [4,10], adherence remains suboptimal in clinical trials [15] and is likely far poorer outside the research context [1,24], suggesting the need for innovative, low-cost interventions to improve medication adherence in both clinical and research contexts [25]. Notably, evidence suggests that more rigorous adherence monitoring is associated with greater medication effects [1], suggesting that monitoring alone could improve adherence.

A number of Internet-based interventions have been developed to address medication adherence for variety of conditions. A review of 13 studies noted that, although some have shown efficacy, only one study used a high-quality measure of adherence [26]. Some form of monitoring was a key component in several of these interventions, and while a few used mobile self-monitoring devices, none used the most ubiquitous and convenient device currently available: the mobile phone. Because mobile phones offer a high degree of access to individuals in their daily lives, they are well suited for just-in-time intervention delivery [27,28].

Initial mobile health (mHealth) interventions for improving medication adherence [29,30] have used text message medication reminders, functioning essentially as an alarm clock. In contrast, the present study incorporated a recently developed platform for mobile, web-based adherence reminders that matches intervention intensity to adherence performance, while also assessing self-reported adherence on an ongoing basis. This low-cost, low-resource, easily scalable mHealth intervention, delivered via smartphone, integrates text message reminders and mobile web-based adherence assessment. The present study is a randomized, controlled trial to evaluate its feasibility and efficacy for improving naltrexone adherence in a treatment-seeking sample [31]. We hypothesized that, compared to a control condition involving mobile self-report of side effects, drinking, and craving, the addition of adaptive mobile medication reminders and adherence assessments would be associated with better adherence, which in turn would be associated with better treatment outcomes.

## Materials and Methods

The protocol for this trial and supporting CONSORT checklist are available as supporting information; see S1 CONSORT Checklist and S1 Protocol.

### Participants

Participants (N = 76) were 21–55 years old. Inclusion criteria included a) average weekly consumption of ≥14 (women) or ≥21 (men) standard drinks over the past 3 months; b) ≥2 heavy drinking days (≥4 [women] or ≥5 [men] standard drinks) in a 30-day period within the prior 90 days; c) desired assistance to reduce or stop drinking alcohol; and d) willingness to take naltrexone. Individuals were excluded for likelihood of pregnancy; opioid use or drug use other than alcohol, nicotine, or cannabis in the prior 30 days; history of opioid dependence or psychotic disorder; use of psychiatric medication other than antidepressants; concurrent treatment for alcohol use other than Alcoholics Anonymous; or a requirement to attend treatment.

### Procedures

The study protocol was reviewed and approved by the University of New Mexico Human Research Review Committee and Quorum Review (Seattle, WA), ClinicalTrials.gov Identifier: NCT01349985.

#### Recruitment and Screening

**Fig 1:**
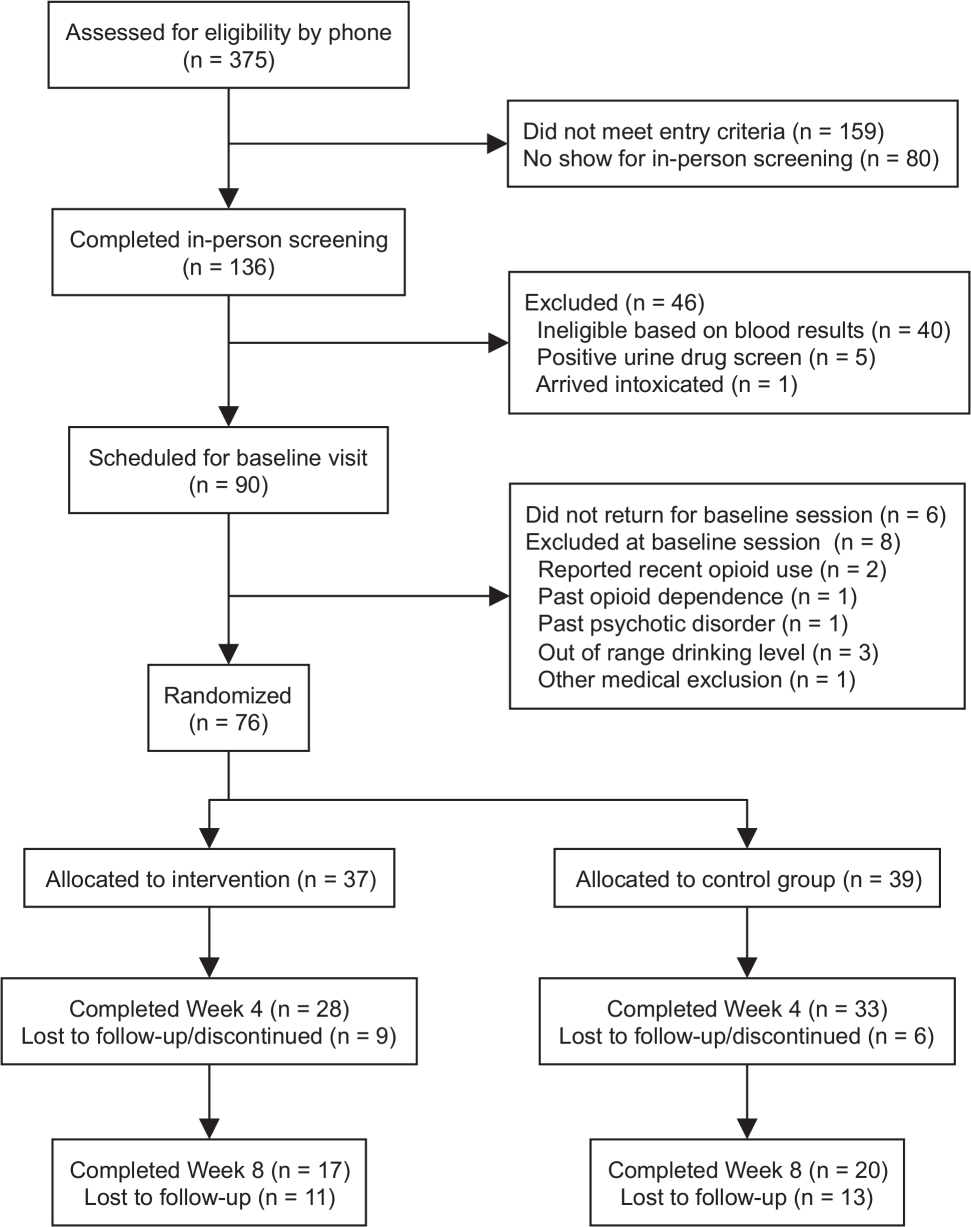
Study CONSORT diagram.

Participants were recruited using newspaper, radio, and online advertisements in the Albuquerque, New Mexico area between August 2011 and November 2012. Advertisements stated that researchers were seeking participants who wished to cut down or quit drinking for involvement in a medication study. Those passing a phone screen were scheduled for further in-clinic screening, at which the study was fully described, any questions were answered, and written informed consent was obtained before continuing. Participants then completed a demographics questionnaire, urine drug toxicology and pregnancy screening, and a blood draw. Participants also completed an interview assessment that included a structured diagnostic interview; all participants met criteria for a current (past year) alcohol use disorder (alcohol abuse or alcohol dependence). The study psychiatrist reviewed blood work results and approved those within acceptable limits. Participants were asked to abstain from alcohol for 72 hours prior to their scheduled baseline appointment to facilitate a clinical assessment of withdrawal symptoms using the Clinical Institute Withdrawal Assessment (CIWA-Ar) protocol. A CIWA score of ≥8 was an additional exclusion criterion. Fig 1 illustrates participant recruitment and flow through the study.

#### Randomization

Participants completed additional baseline questionnaires, after which random assignment to parallel conditions, either the intervention (n = 37) or control (n = 39), occurred 1:1 via a computerized algorithm that balanced groups according to self-reported drinking goal (to moderate or to abstain). Random allocation sequences had been generated in blocks of 8 by computer algorithm before the study began. To conceal the allocation sequence from study personnel, the process was computer-automated. The experimenter was notified via text message (SMS) from the computer of each random assignment as it occurred. Given that the efficacy of naltrexone has been demonstrated and this study examined adherence as the primary outcome, there was no placebo condition.

#### Post-Randomization Procedures

Randomized participants were provided with an initial one-month supply of naltrexone (50mg/day) and an Android smartphone with 8 weeks of unlimited service. Participants were instructed on using the phone to respond to question prompts. All were told to expect daily questions about side effects, alcohol use, and alcohol craving, with the additional possibility of text message reminders and assessments of naltrexone adherence. Participants were instructed to bring their medication bottle upon their return to the clinic at Weeks 4 and 8.

In-clinic assessments at Weeks 4 and 8 included an assessment of side effects and past 30-day alcohol use, a blood draw, and a physician follow-up assessment. Any smartphone-related problems were addressed. Those medically approved to continue received a medication refill. Participants were compensated at each visit and allowed to keep the study smartphone. No changes were made to the methods or primary outcomes after the trial commenced. At Week 8, those interested in continuing naltrexone received referrals. Additional study protocol details have been reported previously [31].

#### Intervention Condition

The intervention was called AGATE-Rx (Adaptive, Goal-directed Adherence Tracking and Enhancement). Its design and development is described elsewhere [31]. In brief, AGATE reminded participants to take the study medication via SMS text messages. Additionally, a hyperlink in the message launched the web browser to assess adherence. Over time, the frequency of medication reminders was adapted according to self-reported adherence performance (see supplemental material for details). In addition to the AGATE-Rx adherence reminders/assessments, participants in the intervention condition received a Smartphone Alcohol and Side Effects Diary (SASED), consisting of daily SMS prompts with hyperlinks to assess side effects and alcohol use and craving. Screenshots from the intervention and content of all SMS messages and assessments are available as supporting information; see S1 Screenshots and S1 Content.

#### Control Condition

To maximize similarity across conditions with respect to frequency of reporting on side effects and alcohol-related variables, as well as demand characteristics implicit in receiving a prepaid smartphone, participants in the control condition received a prepaid phone and received SASED prompts/assessments, but not adherence reminders/assessments.

### Measures

#### Structured Clinical Interview for DSM-IV-TR (Baseline)

Sections covering diagnostic criteria for substance use disorders and psychosis were administered [32].

#### Alcohol Use Disorders Identification Test (AUDIT, Baseline)

The AUDIT [33] is a validated 10-item measure of alcohol involvement. Items are summed to create a score (ranging 0–40).

#### Alcohol Dependence Scale (ADS, Baseline)

The ADS [34] is a 25-item scale assessing alcohol dependence during the past 12 months. Items are summed to yield a quantitative index (ranging 0–47).

#### Penn Alcohol Craving Scale (PACS, Baseline)

The five items of the PACS [35] concern the frequency, intensity, and duration of thoughts about drinking; the ability to resist drinking; and average craving over the past week. Items were averaged to yield a score ranging 0–6. Part of the SASED, the craving intensity item ("At its most severe point, how strong was your craving to drink alcohol?") was re-administered daily, referring to the preceding day.

#### Alcohol use

The Timeline Follow-back (TLFB) calendar method [36] was used to quantify alcohol use during the 90 days preceding Baseline and the 28 days preceding Weeks 4 and 8. As part of the SASED, drinking was queried daily via smartphone. Participants were asked how many standard drinks they consumed on the prior day, capped at 13 drinks.

#### Side Effects

As part of the SASED, side effects were queried daily via smartphone.

#### Medication Event Monitoring System (MEMS, MWV Healthcare)

The MEMS is a system that integrates a small microcircuit into a lid for a medication vial, which records the time and date whenever the lid is opened. MEMS monitors are capable of storing up to 3800 medication events. According to the developers, over 700 peer-reviewed publications have used MEMS to compile drug dosing histories and assess compliance in patients in a wide variety of clinical situations (http://www.mwvaardex.com). Thus, MEMS is considered by many to be the "gold-standard" test of the reliability of medication-event monitoring. In the present study, participants received medication in a standard pill bottle with an electronic, chip-embedded MEMS cap that logged all openings. We attempted to retrieve MEMS caps from all randomized participants; however, some caps could not be retrieved. Raw MEMS adherence data were downloaded from 60 returned caps.

### Sample Size Determination and Specification of Outcomes

As specified in the study protocol [31] our primary outcome was the proportion of participants with adequate adherence, defined as at least 80% of prescribed doses taken during the eight-week trial, as measured by MEMS. Details on the sample size determination can be found in the study protocol [31]. In brief, a similar study by Simoni et al. [20] found a medium effect size for a pager intervention compared to a peer support intervention (*w* = .36) and compared to usual care (*w* = .32) using chi-square tests. Therefore, we anticipated a medium effect size for AGATE compared to SASED. Power analysis was conducted using G*Power 3 software [37,38]. For a χ² test with d.f. = 1 and α error probability set to. 05, a sample size of 88 yields power of. 80 to detect an effect size *w* = .30. Due to funding and time constraints, enrollment ended when the number of randomized participants reached 76.

Secondary outcomes were the continuous measures of adherence (i.e., percent of doses taken), latency to fall below 80% of cumulative doses taken, drinks per drinking day, craving intensity, latency to first drinking day (for those with an abstinence goal), and latency to first day of heavy drinking (≥4 [women] or ≥5 [men] standard drinks).

### Statistical Analysis

Preliminary analyses included T-tests and chi-square tests to examine whether 1) randomized participants differed from non-randomized participants in terms of demographics (age, gender, race, ethnicity, education level, or income); 2) Week 4 and Week 8 attendees differed from non-attendees in terms of demographics, drinking goal, AUDIT score, and ADS score; and 3) experimental conditions differed with regard to demographics, drinking goal, MEMS cap return, side effects, Week 4 and Week 8 attendance, and numbers of smartphone assessments completed. Indices of side effects included the proportion reporting any side effects in Days 1–7, 1–28, and 1–56, as well as a side effects score comprising the total number of side effects multiplied by number of days endorsed during each of these periods. Pearson's correlations were used to examine whether the indices of side effects were related to indices of adherence at mid-study or study end.

Given the once per day dosing schedule for naltrexone, MEMS data were cleaned to ensure that no more than one opening per day was counted as a taken dose. For each participant, the number of doses taken was expressed as a proportion of number of openings divided by number of days for Days 1–28 and Days 1–56. Each participant was then dichotomously coded as below or above our *a priori* 80% adherence threshold at mid-study and over the full 56 days.

The primary analysis examined the proportion of patients who were categorized as adherent (i.e., ≥80% of cumulative doses taken), All randomized participants were included in this analysis. If the MEMS cap was not returned, overall adherence was counted as zero. Secondary analyses examining continuous adherence data included both intention-to-treat (ITT) and per-protocol analyses. For ITT, overall adherence was computed for all randomized participants as a proportion of days on which an opening occurred from Day 1–56, unless the MEMS cap was not returned, in which case adherence was coded as 0%. Adherence was examined using analysis of covariance (ANCOVA) in the form of hierarchical multiple regression. Variables differing between groups at baseline were entered as covariates on the first step of hierarchical multiple regression analyses. R-square change was evaluated as experimental condition was entered into the regression equation on the second step of the hierarchical analysis. The per-protocol analyses focused on Week 4 outcomes for two reasons. First, participants who did not return for the Week 4 visit did not receive a refill and thus could not adhere for the full 56-day period. Second, the number of participants retained through Week 8 (n = 37) was substantially lower than the number required to detect a significant intervention effect on the primary outcome [31], reflecting high attrition. Given inadequate power to address the primary hypothesis with the Week 8 outcomes, unplanned per-protocol analyses examined mid-study adherence among those attending Week 4 whose MEMS caps were returned (n = 60), excluding 2 participants who had disclosed information invalidating their MEMS data (discarding pills, taking half- or double-doses).

Given that adherence tends to decline over time, analyses also examined whether the intervention helped participants maintain adequate adherence in the initial weeks of treatment. A secondary (unplanned) survival analysis examined latency to fall below 80% of cumulative doses taken. Daily cumulative adherence was defined as cumulative number of days with a bottle opening divided by cumulative number of days in the trial. Kaplan-Meier survival analysis evaluated whether experimental conditions differed in latency to fall below 80% adherence at midstudy and at study end.

To evaluate the association of adherence with relapse to heavy drinking, Kaplan-Meier survival analysis compared those above and below the adherence threshold at mid-study (80% doses taken, collapsed across experimental condition) with regard to two relapse variables derived from the Week 4 TLFB: latency to first drinking day (for those with an abstinence goal) and first day of heavy drinking (≥4 [women] or ≥5 [men] standard drinks).

Smartphone alcohol drinking and craving data were aggregated across Days 1–28 and 29–56 and compared using t-tests. All participants making at least one report during the 28-day period received a mean score for that period. SASED-derived mean drinks per drinking day during Days 1–28 and 29–56 were compared with TLFB-derived drinks per drinking day for the same periods using Pearson's correlations.

## Results

### Sample Characteristics

Table 1 presents baseline demographic and clinical characteristics of randomized participants. No differences were found between randomized and non-randomized individuals and between Week 4 attendees and non-attendees (study dropouts). Compared to Week 8 attendees, non-attendees (study dropouts and medical withdrawals/exclusions) were older (39.7 [SD = 8.9] vs. 35.5 [SD = 9.5] years, t[74] = -2.00, p = .049) and more likely to be female (51.2% vs. 16.2%, *χ²*[1] = 10.37, p = .001). Despite random assignment, the conditions differed with regard to gender, income, and ADS score; therefore, these variables were used as covariates. The experimental conditions did not differ on attendance, MEMS cap returns, or reported side effects.

**Table 1:** Baseline Demographic and Clinical Characteristics of Randomized Participants.

			Treatment Group					
	Overall		Intervention		Control			
	(N = 76)		(n = 37)		(n = 39)			
	**M**	**SD**	**M**	**SD**	**M**	**SD**	**t(74)**	**p**
Age (years)	37.5	9.4	39.6	9.6	35.6	8.8	-1.90	.062
Drinks Per Drinking Day*	10.2	5.6	9.0	4.4	11.3	6.4	1.76	.082
Percent Drinking Days*	69.3	25.0	72.8	25.1	65.9	24.7	-1.21	.230
Penn Alcohol Craving Scale	3.1	1.4	3.0	1.4	3.2	1.4	0.39	.701
Alcohol Use Disorders Identification Test	23.0	7.6	22.4	7.9	23.6	7.4	0.67	.503
Alcohol Dependence Scale	16.0	8.4	13.9	7.5	18.0	8.8	2.15	.035
	**N**	**%**	**N**	**%**	**N**	**%**	**χ²(1)**	**p**
Sex (female)	26	34.2	18	48.6	8	20.5	6.68	.010
Race (White, non-Hispanic)	27	35.5	16	43.2	11	28.2	1.88	.171
Ethnicity (Hispanic)	37	48.7	20	54.1	17	43.6	0.83	.362
Partnered (married, engaged, or cohabiting)	30	39.5	17	45.9	13	33.3	1.26	.261
Education (Bachelor's degree or higher)	14	18.4	9	24.3	5	12.8	1.67	.196
Annual Income ($20,000 or higher)	41	53.9	25	67.6	16	41.0	5.39	.020
DSM-IV-TR Alcohol Dependent	52	70.3	25	69.4	27	71.1	0.23	.880
Drinking Goal (Abstain)	40	52.6	19	51.4	21	53.8	0.47	.828

*Drinking variables were computed for preceding 90 days using the Timeline Followback Questionnaire.

### Sensitivity Analysis

Because our sample size of N = 76 fell short of our enrollment goal of 88 participants, we conducted a sensitivity analysis to determine the effect size that could be detected with a χ² test with d.f. = 1, α error probability set to. 05, and power of. 80. The analysis, conducted using G*Power 3 software [37,38], indicated that the detectable effect size with those parameters was *w* = .32, which was within the range observed by Simoni et al. [20].

### Smartphone Engagement

Over Days 1–56, control participants averaged 39.2 [SD = 15.6] responses to SASED assessments. Intervention participants averaged 28.6 [SD = 16.0] SASED responses and 14.4 [SD = 7.5] AGATE-Rx responses for a total average of 42.9 [SD = 22.8]; therefore, the total number of responses did not differ between groups (t[74] = -0.84, p = .404).

### Side Effects and Adherence

Two thirds (67.1%) of participants reported side effects in the first week; one third (33.3%) reported anxiety, 22.7% reported nausea, 18.7% reported dizziness, 30.7% reported thirst, 37.3% reported sleep effects, and 20.0% reported weakness. By Day 28, 78.9% of participants had reported side effects. By study end, 80.3% had reported side effects. No index of side effects correlated significantly with adherence at mid-study or study end.

### Adherence Outcomes by Condition

A chi-square test comparing groups in terms of the proportion of patients with adequate adherence (defined *a priori* as ≥ 80% of doses taken [13,16] was not significant at Week 4, *χ²*[1] = 1.27, p = .260, or at Week 8, *χ²*[1] = .91, p = .341. At Week 4, 48.6% of the experimental group and 35.9% of the control group were categorized as adequately adherent. These numbers assume 0% adherence for participants who did not return MEMS caps. At Week 8, 18.9% of the experimental group and 30.8% of the control group were categorized as adherent. As shown in Table 2, a secondary analysis examining adherence as a continuous outcome showed that experimental condition did not predict overall or mid-study adherence. Controlling for the covariates, mean overall adherence over 8 weeks was. 500 [95% CI = .381–.618] in the experimental group and. 453 [95% CI = .338–.568] in the control group. These means include zeros for 16 participants who did not return MEMS. Experimental condition also did not predict mid-study adherence in the per-protocol analysis including those whose MEMS caps were returned at Week 4. Controlling for covariates, mid-study adherence was. 828 [95% CI = .742–.915] in the experimental group and. 767 [95% CI = .680–.853] in the control group.

**Table 2:** Coefficients from Intent-to-Treat (ITT) and Per-Protocol (PP) Analysis of Adherence.

**Dependent Variable: Overall Adherence (ITT)**		**β**	**t**	**p**	**ΔR²**	**F**	**df**	**p**
Step 1 Variables Entered		–	–		.053	1.34	3, 72	.267
	Sex (female)	-.195	-1.70	.093	–	–	–	–
	Annual Income ($20,000 or higher)	.008	0.07	.944	–	–	–	–
	Alcohol Dependence Scale	-.113	-0.97	.335	–	–	–	–
Step 2 Variables Entered		–	–	–	.004	0.29	1, 71	.594
	Experimental Condition	.069	0.54	.594	–	–	–	–
**Dependent Variable: Midstudy Adherence (PP)**		**β**	**t**	**p**	**ΔR²**	**F**	**df**	**p**
Step 1 Variables Entered		–	–	–	.012	0.21	3, 50	.890
	Sex (female)	.076	0.5	.591	–	–	–	–
	Annual Income ($20,000 or higher)	.079	0.6	.582	–	–	–	–
	Alcohol Dependence Scale	.048	0.4	.736	–	–	–	–
Step 2 Variables Entered		–	–	–	.018	0.89	1, 49	.350
	Experimental Condition	.155	0.9	.350	–	–	–	–

**Fig 2:**
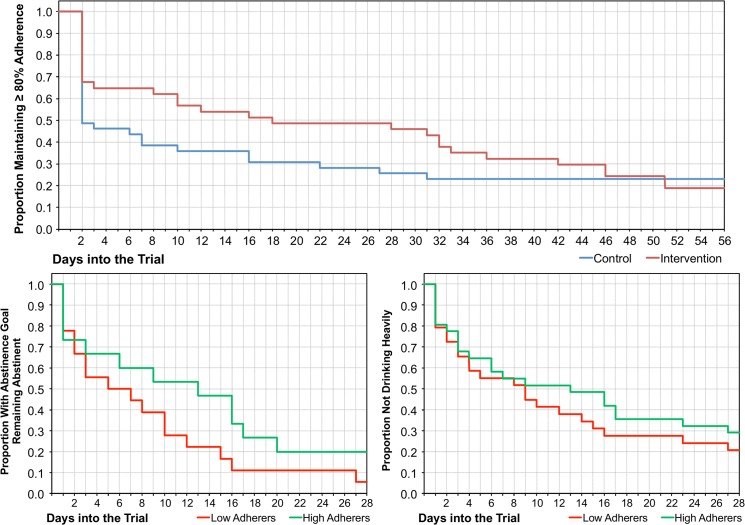
Study outcomes. Time to first drop below 80% cumulative adherence among all randomized participants, time to first drink among participants with a goal of abstinence completing the Week 4 Timeline Followback assessment, and time to first heavy drinking episode among all participants completing the Week 4 Timeline Followback assessment.

As illustrated in Fig 2, the secondary survival analysis revealed that the time to drop below 80% adherence was significantly different between experimental groups at midstudy (Mantel-Cox *χ²*[1] = 4.28, p = .039) but not at study end (Mantel-Cox *χ²*[1] = 0.46. p = .497). The median latency to fall below 80% cumulative adherence was 19 days [95% CI = 0.0–44.0] in the intervention group and 3 days [95% CI = 0.0–8.1] in the control group.

### Alcohol Use Outcomes by Condition

**Fig 3:**
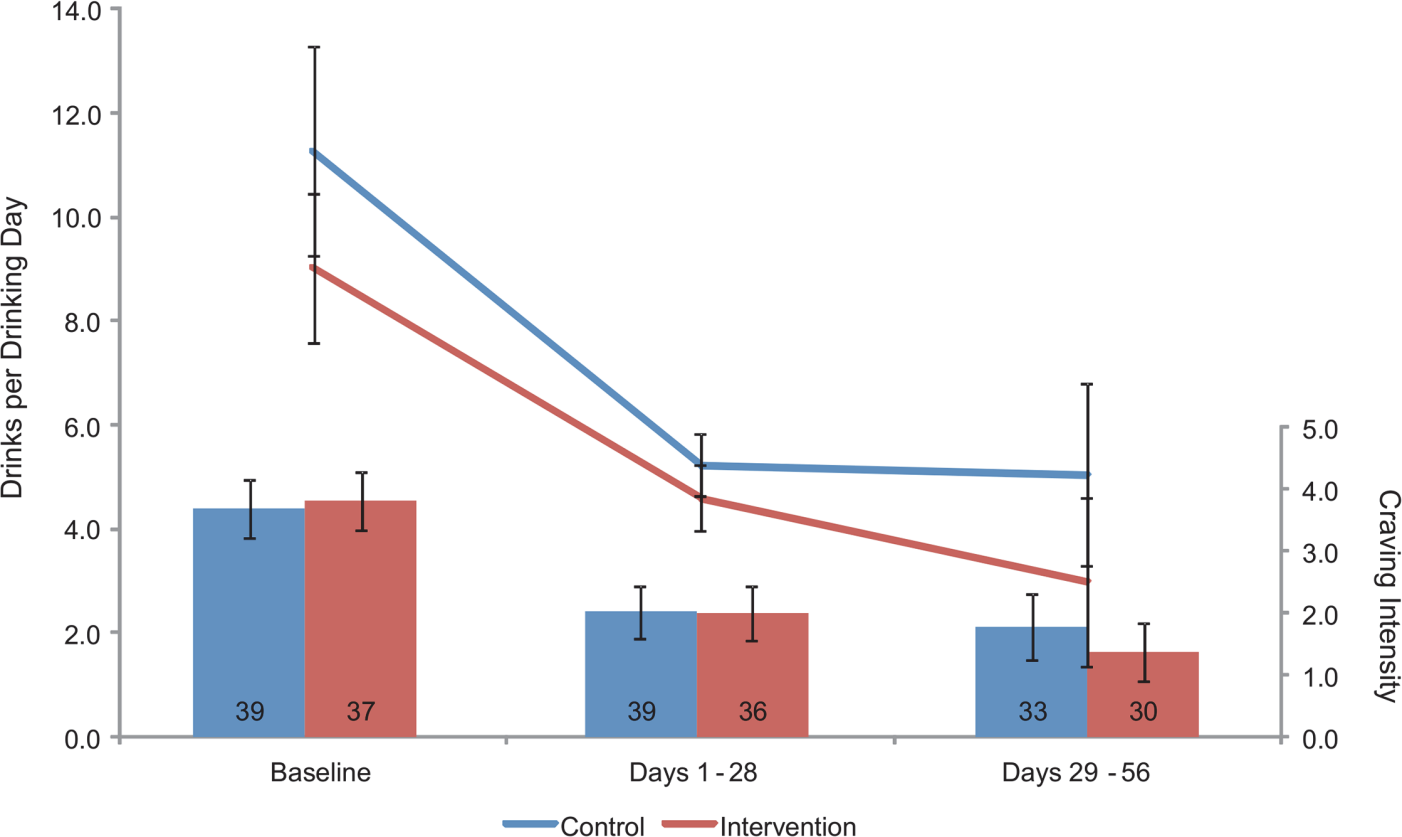
Changes in Self-Reported Drinking and Craving. 95% confidence intervals around the mean numbers of self-reported drinks per drinking day (line graph) and ratings of alcohol craving intensity (column graph), assessed via smartphone, among heavy drinkers randomly assigned to the intervention or control condition. The number of participants making at least one report for each time frame is the number shown in the columns.

Fig 3 depicts 95% confidence intervals over time for drinks per drinking day and craving intensity, assessed via daily smartphone prompts. Across conditions, craving intensity decreased from Baseline [M = 3.8, SD = 1.5] through Days 1–28 [M = 2.0, SD = 1.3] (t[74] = 9.2, p<.001) and 29–56 [M = 1.6, SD = 1.5] (t[62] = 10.4, p<.001). The experimental conditions did not differ. Similarly, across conditions, drinks per drinking day decreased from Baseline [M = 10.0, SD = 5.7] through Days 1–28 [M = 4.9, SD = 1.8] (t[62] = 7.4, p<.001) and 29–56 [M = 4.1, SD = 4.5] (t[52] = 6.6, p<.001), with no difference between conditions. For comparison, mean drinks per drinking day derived from the TLFB were 2.8 [SD = 1.8] (Week 4) and 2.2 [SD = 1.4] (Week 8). Correlations between smartphone and TLFB measures of drinks per drinking day were. 430 (p = .001, n = 52) for Week 4/Days 1–28 and. 516 (p = .008, n = 25) for Week 8/Days 29–56.

#### Drinking Outcomes Stratified by Adherence

Fig 2 shows drinking outcomes stratified by midstudy adherence. Compared to those with <80% adherence (n = 29), those with ≥80% adherence (n = 31) did not significantly delay heavy drinking (Mantel-Cox *χ²*[1] = 0.68. p = .408). Among those whose goal was to abstain, compared to those with <80% adherence (n = 18), those with ≥80% adherence (n = 15) did not significantly delay drinking (Mantel-Cox *χ²*[1] = 2.11. p = .146).

## Discussion

This study is the first randomized, controlled trial to evaluate a mobile health (mHealth) intervention designed to improve adherence to pharmacotherapy in the treatment of alcohol use disorders. Results did not support the hypothesis that text message medication reminders and mobile web-based adherence assessment would improve adherence, above and beyond a control condition consisting of mobile assessment of side effects, drinking, and craving. Notably, high attrition resulted in an insufficient sample size at Week 8. In this context it was not surprising that ITT analyses (including all randomized individuals and assigning 0% adherence to those who discontinued) revealed no intervention effect and low overall estimates of adherence (50% cumulative through Week 8). A per-protocol analysis through Week 4 showed adherence rates of 83% (intervention group) and 77% (control group). These rates approach those reported in studies using psychosocial interventions to maximize adherence [14,15].

Secondary analyses suggested that the intervention group maintained adequate adherence (≥80% of doses taken) longer than the control group through Week 4, although the effect was not observed through Week 8. Therefore, one possibility is that mHealth approaches for monitoring adherence (or otherwise maintaining contact with participants) can support adequate short-term adherence in cases where psychosocial interventions are not available. Taken together, these findings suggest that a smartphone intervention involving daily assessment, without or without medication reminders, warrants further evaluation for maintaining adherence to pharmacotherapy among those engaged in treatment. The results further suggest that daily adherence reminders and assessments via smartphone may help sustain adherence during the first weeks of naltrexone treatment, when the incidence of relapse is generally highest. Contrary to other studies, adherence to naltrexone (defined as ≥80% of doses taken) was not associated with significantly longer latencies to first heavy drinking day (for all participants) or first drinking day (among those with an abstinence goal).

Advantages of the current study include the use of a novel technology designed specifically to support medication adherence during treatment for alcohol use disorders, and the use of MEMS to provide a relatively objective measure of adherence. While MEMS is a widely accepted measure of adherence, it indicates bottle openings rather than actual medication use; having a biomarker of adherence would increase confidence in pill adherence results. The comparison condition sought to control for the possibility that receiving a smartphone and/or using it to perform self-monitoring would exert an effect. The control condition was both a strength and a limitation, as it did not enable us to examine the effect of the intervention compared to treatment-as-usual (i.e., treatment without daily assessments via smartphone). The difference in attention (i.e., the number of text messages) between the conditions could be viewed as a limitation. Other limitations include the short 8-week treatment protocol and a relatively small sample size, in part reflecting the high number of study dropouts and discontinuations between Week 4 and Week 8. Attrition limited the statistical power and utility of the Week 8 assessments. Finally, while secondary analyses indicated that participants in the intervention condition maintained ≥80% adherence significantly longer than control participants, the clinical significance of this outcome has not been validated, and there was no association of medication adherence with drinking outcomes in the current sample.

This study offers preliminary support for the feasibility of mHealth interventions among treatment-seeking participants with alcohol use disorders. Participant engagement was relatively good in both conditions, with a number of smartphone assessments completed over the course of the study, which addresses concerns that individuals with alcohol dependence would be unlikely to complete mobile assessments [25]. We expected that accountability for medication adherence via smartphone reminders and assessments improve adherence above and beyond daily alcohol use assessments; however, participants who were merely prompted to report on side effects, drinking, and craving had similar adherence rates as the intervention group, possibly through self-monitoring of the very targets of the medication: drinking and craving. It is important to acknowledge, however, that we did not have a reminder-without-assessment condition, which would be necessary to evaluate how mobile assessment per se compares to adherence reminders alone.

Evidence that remote monitoring of adherence and/or alcohol use improves treatment engagement would have implications for real-world treatment settings given the low cost of these interventions. All participants received smartphones with unlimited service to use in the study, costing approximately $300 USD per participant. We did not give participants an option to use their own smartphones so as to standardize and control the intervention experience. When such standardization and control are not concerns, intervention cost would be reduced as individuals across socioeconomic classes increasingly already have smartphones [39]. Nonetheless, prepaid smartphones and service plans are decreasing in cost and may provide a more cost-effective than psychosocial interventions, which require in person attendance with a trained professional. mHealth interventions have additional advantages of requiring minimal staff time and enabling daily contact. Although not used in the current study, feedback loops can be incorporated to alert staff under predetermined conditions, for example, if certain side effects are reported or adherence has not been reported for a period of time.

In summary, the present findings support the feasibility of implementing a mHealth intervention for supporting adherence to pharmacotherapy among treatment-seeking participants; however, the current results did not support the efficacy of text message reminders on adherence, above and beyond remote assessments of drinking and craving. Despite these findings, trials with larger samples and alternate designs are warranted. In particular, given the use of a relatively rigorous control condition in the present study, future studies should compare mobile assessments and reminders to treatment-as-usual. Further questions include whether mHealth interventions can achieve adherence rates comparable to those achieved with psychosocial adherence interventions, or whether mHealth interventions could enhance effects of psychosocial interventions.

## Supporting Information

S1 CONSORT Checklist (PDF)

S1 Content Study Smartphone Message and Assessment Content. (PDF)

S1 Protocol Study Protrocol. (PDF)

S1 Screenshots Screenshots from the Control and Intervention Conditions. (PDF)
