# Supplementary material for: Randomized Controlled Trial of a Mobile Phone Intervention for Improving Adherence to Naltrexone for Alcohol Use Disorders
Source: PLoS One. 2015 Apr 24;10(4):e0124613. doi: 10.1371/journal.pone.0124613 (PMC4409303; doi:10.1371/journal.pone.0124613)
Supplement: S1 Content — (PDF) [file pone.0124613.s002.pdf]

SUPPLEMENTAL TABLE 1. Study Smartphone Message and Assessment Content

Both Intervention and Control Conditions: Daily Assessment of Side Effects, Drinking, and Alcohol Craving (SASED)

§ "Hi [Nickname]. Couple of quick questions for you. Please click this link: http://[web address]"

- "Hi [Nickname]! A couple of quick questions… Are you having any side effects? No, Yes/Unsure"
    - Yes/Unsure: "Get emergency medical help if you have signs of an allergic reaction: hives, difficulty breathing, or swelling of your face, lips, tongue, or throat. Call your doctor if you have any of these serious side effects: blurred vision or eye problems, fast heart beat, mood changes, hallucinations, confusion, thoughts of hurting yourself, severe nausea, stomach pain, low fever, loss of appetite, dark urine, clay-colored stools, yellowed skin or eyes, ear pain or ringing, skin rash or itching, wheezing, or difficulty breathing."
        - "Are you having any of the following (please check all that apply): nausea, anxiety, light-headedness, increased thirst, fatigue or weakness, sleep problems, other (specify)."
- "At its most severe point, how strong was your craving for alcohol yesterday? None at all…Strong urge and would have drunk alcohol if it were available."
- "Did you drink any alcohol yesterday? No, Yes"
    - Yes: "How many 'drinks' of alcohol did you have yesterday (1 'drink'=12 oz beer=5 oz wine=1 oz liquor)?
      Less than 1 drink, 1 drink, 2 drinks,…13 or more drinks"

- "Thanks [Nickname]! Have a great day!"

Intervention Condition Only: Stage 1 Daily Reminders/Assessments of Adherence (AGATE-Rx Stage 1)

§ "Hi [Nickname]. Don't forget your med today. Please tell us how you're doing: http://[web address]"

- "Hi [Nickname]! Time for your [time] meds. Click here for details and instructions if you need them.
- Please click here to tell us about your last dose."
    - "Yesterday your study medication was scheduled for [time]. Did you take your study medication yesterday? Yes, No"
        - Yes: "Nice job! Keep up the good work!"
        - No: "Please select the one reason that best describes why you did not take your medication on time."
            - "Thanks! Please don't be discouraged about yesterday. Today is a new day! Just do whatever you can to take all your medication on time today. AGATE-Rx will try to help you do your best."

Intervention Condition Only: Stage 1 Evaluation of Daily Adherence Self Reports, ≥80% adherence (>5 of 7 responses are "yes")

§ "Hi [Nickname]. You're doing great with daily meds! Please keep it up. We'll back off with reminding and only check in every 3 days."

Intervention Condition Only: Stage 2 Reminders/Assessments of Adherence Every 3rd Day

§ "Hi [Nickname]. How's it been going with your meds? Please let us know: http://[web address]"

- "Have you taken your study medication every day for the last 3 days? Yes, No"
    - Yes: "Nice job! Keep up the good work!"
    - No: "Please select the one reason that best describes why you did not take your medication on time."

Intervention Condition Only: Stage 2 Biweekly Evaluation of Adherence Self Reports, <80% adherence (<4 of 4 responses are "yes")

§ "Hi [Nickname]. Seems you're not taking your med often enough. Let's go back to daily reminders to help you get back on track."

Both Intervention and Control Conditions: Attempt to Answer an Assessment That Has Already Been Answered

"Our records show you already responded to this message. Thanks!"

Both Intervention and Control Conditions: Attempt to Answer an Assessment After 24 Hours

"We're sorry, this message is expired. If you want to respond, please select a more recent message."

Note: § indicates content was delivered via text message. All other content was delivered via mobile web browser on the smartphone. To limit retrospection, all assessments expired in 24 hours from when they were sent, and each hyperlink worked only once. Responses were requested but not incentivized. In Stage 1, patients received one reminder daily with a hyperlink to report medication adherence the previous day. After 7 days, if participants reported ≥80% adherence, they advanced to Stage 2 with reminders and assessments occurring every 3rd day. If not, self-reported adherence was re-evaluated after 7 days. In Stage 2, self-reported adherence was evaluated after 14 days. Those with <80% adherence reverted to Stage 1.
