# Supplementary material for: Randomized Controlled Trial of a Mobile Phone Intervention for Improving Adherence to Naltrexone for Alcohol Use Disorders
Source: PLoS One. 2015 Apr 24;10(4):e0124613. doi: 10.1371/journal.pone.0124613 (PMC4409303; doi:10.1371/journal.pone.0124613)
Supplement: S1 Protocol — (PDF) [file pone.0124613.s003.pdf]

Study Protocol

I. Research Project Title

AGATE: Adaptive Goal-Directed Adherence Tracking for Naltrexone

II. Investigator's Name, Degree, Title, and Department

Christian Hendershot Ph.D.
Research Scientist
The Mind Research Network and
Center on Alcoholism, Substance Abuse and Addictions (CASAA), UNM

III. Hypothesis or Study Goals (questions hoped to be answered by study):

The overall goal of the study is to evaluate the efficacy of a new smartphone-based technology for tracking and enhancing medication adherence. The technology, AGATE (Adaptive Goal-Directed Adherence Tracking and Enhancement), was recently developed by our colleagues at Talaria, Inc. (Seattle, WA) for the purpose of enhancing medication adherence for individuals with alcohol problems. In the current study we will test the efficacy of AGATE for increasing medication adherence in an eight-week pharmacotherapy study of naltrexone. Participants will be randomly assigned to smartphone-based adherence support (AGATE) or a control condition (smartphone alcohol and side effects diary, SASED). A secondary goal is to examine medication effects on patterns of daily drinking using ecological assessments permitted by cell-phone assessments.

The specific hypotheses are as follows:

Hypothesis 1: Those assigned to the AGATE condition will be more likely to be classified as adherent to naltrexone (e.g., taking 80% or more of scheduled doses) over the course of the study compared to those assigned to SASED.

Hypothesis 2: Those in the AGATE condition will take significantly more scheduled doses compared to those in SASED.

Hypothesis 3: Those in the AGATE condition will show better drinking outcomes (defined as higher percentage of days abstinent, lower percentage of heavy drinking days, fewer drinks per drinking day, and longer latency to first heavy drinking day) compared to SASED.

Hypothesis 4: Those in the AGATE condition will report higher satisfaction with treatment compared to SASED.

Hypothesis 5: Medication adherence will moderate the effect of naltrexone on drinking outcomes, such that the effects of naltrexone on reduced heavy drinking will be increased among those with higher rates of adherence.

Hypothesis 6: Adherence outcomes will mediate the effects of condition assignment (AGATE vs. SASED) on drinking outcomes.

IV. Background:

i) Describe relationship of proposed study to previous investigations in the field, summarize those previous studies, including previous human, laboratory, and animal studies (describe existing knowledge).

Non-adherence to prescribed medications remains a significant and persistent barrier to the successful management of chronic medical conditions. It is estimated that non- or sub-optimal adherence accounts for 33-69% of medication-related hospital admissions at an annual cost of $100 billion to the U.S. health care system (Osterberg & Blaschke, 2005). Non-adherence not only undermines treatment effectiveness on an individual basis but can also pose a threat to public health by promoting the development of drug-resistant pathogens (Paterson et al., 2000). The intransigent nature of non-adherence is illustrated by the modest adherence rates observed under even optimal conditions: clinical trials designed to maximize medication compliance among patients with chronic conditions have reported adherence rates ranging from 43 to 78% (Osterberg & Blaschke,

2005).

Alcohol use has been established as a significant factor in adherence to complex medication regimens (Galvan et al., 2002; Hendershot et al., 2009; Samet et al., 2007). Studies examining multiple levels of drinking (e.g., any vs. moderate vs. heavy use) have reported a linear relationship between drinking severity and nonadherence (Braithewaite et al., 2005, Samet et al., 2004; Tucker et al., 2003). Additionally, studies have linked temporal increases in patient drinking with greater non-adherence (Braithwaite et al., 2005; Spire et al., 2002). Finally, a recent meta-analysis confirmed the dose-response association of alcohol use with medication nonadherence in an analysis of 40 published studies totaling over 25,000 participants (Hendershot et al., 2009). With few exceptions (e.g., Parsons et al., 2007; Samet et al., 2004), medication adherence interventions have seldom focused specifically on individuals with alcohol use disorders (AUDs). Importantly, while three FDA-approved medications exist for treating alcohol use disorders (naltrexone, acamprosate, disulfiram), nonadherence is a specific barrier to the efficacy of these medications, partly due to the association of alcohol use with medication non-adherence. Given this barrier, further research is needed to a) investigate patterns and correlates of adherence to pharmacotherapy among those with alcohol use disorders, and b) to maximize medication adherence among heavy drinkers who are initiating pharmacotherapy.

Naltrexone is one of three FDA-approved medications for alcohol dependence. Naltrexone not only shows efficacy for reducing levels of alcohol use in alcohol-dependent individuals (Anton et al., 2006) but appears to reduce heavy drinking in non-dependent individuals with high-risk drinking patterns (Kranzler et al.,2003). However, naltrexone is only effective for some individuals and adherence to naltrexone has been identified as a key barrier to maximizing treatment efficacy. Because naltrexone is a front-line pharmacotherapy for alcohol dependence, the identification and remediation of barriers to naltrexone adherence could potentially lead to improved clinical outcomes in a substantial number of heavy-drinking individuals.

Feinn et al. (2003) conducted a well-designed study to evaluate multiple methods of measuring medication adherence in the context of naltrexone treatment for problem drinking. One hundred fifty heavy drinkers ($\geq$ 24 standard drinks per week for men, and $\geq$ 18 standard drinks for women) were randomly assigned to receive naltrexone (50 mg once per day) or placebo for 8-weeks. Adherence was measured in all participants using electronic medication electronic monitoring system (MEMS; which records opening of a medication bottle); biweekly pill count, and self-reports using paper-and-pencil diaries. The highest rate of medication adherence was indicated by the diaries (M = .908, SD = .146), followed by MEMS (M = .873, SD = .170), and pill count (M = .857, SD = .170). The authors concluded that the daily diary method provided adherence data that was similar to that provided by the MEMS and that pill counts were less useful than either of the other approaches. AGATE is, in part, an electronic diary of adherence. Therefore, we have chosen to follow Feinn et al.'s design, with the exception of not including a placebo condition (our goal is not to evaluate efficacy of naltrexone versus placebo because this has already been demonstrated).

ii) Identify specific knowledge gaps which research is intended to fill (rationale for performing the research).

Our colleagues at Talaria, Inc. have developed a new medication adherence tracking system, AGATE (Adaptive Goal-directed Adherence Tracking and Enhancement). AGATE is designed to measure and maximize medication adherence and is delivered using smartphone technology. Talaria, Inc recently completed a Phase I Small Business Innovation Research (SBIR) trial (funded by the National Institute on Alcohol Abuse and Alcoholism, NIAAA) to develop AGATE as a method of tracking and enhancing medication adherence among individuals with alcohol problems. During that trial, Dr. Stoner and colleagues developed a prototype of the AGATE system and conducted usability testing. Subsequently, NIAAA invited researchers at Talaria to submit a Phase II SBIR

proposal, which was funded in October 2010. The Phase Ii SBIR study will be a joint effort between Talaria, the Mind Research Network and UNM. The goal of the Phase II SBIR trial is to examine the efficacy of AGATE in the context of a trial of naltrexone for alcohol problems. The goal of the trial is to evaluate the efficacy of AGATE in the context of a pharmacotherapy trial that approximates a real-world treatment setting.

V. Experimental Design and Methods:

i) Provide concise description of experimental design and procedures to be used to accomplish study goals. Include precise descriptions of all tests and measurements, and their expected duration. Include optional testing and blinding/un-blinding procedures if applicable.

Overview of Naltrexone Trial
The purpose of the study is to evaluate whether AGATE effectively measures and enhances medication adherence in the context of a naltrexone trial involving heavy drinkers. Participants will be heavy drinkers recruited from the greater Albuquerque area who are interested in either reducing or stopping their drinking and deemed to be candidates for naltrexone pharmacotherapy by the study physician, Dr. Pamela Arenella. Those enrolled in the study will be prescribed naltrexone (at FDA-approved dose of 50mg once daily) for eight weeks. All participants will receive smartphones during the study. Participants will be randomly assigned to receive either AGATE (adherence support) or SASED, a web-based alcohol and side-effects diary via smartphone. The primary outcome will be percent of scheduled doses that were taken during the eight week trial, as measured by the Medication Event Monitoring System (MEMS, Aardex Group, Union City, CA), pill counts, and the timeline follow-back (TLFB) method. The secondary outcomes will be selected indicators of alcohol use over the 8-week trial.

Procedures. Potential participants will screened by phone to determine basic eligibility criteria (i.e., criteria related to drinking/drug use, pregnancy and medications, etc., as outlined below). In most cases, participants will be responding to flyers, etc. and will call the research office. As noted above, we will also initiate contact with some former participants who had agreed to future contact. Those meeting basic eligibility criteria on the phone screening will be scheduled for an in-person screening visit. At the in-person screening visit participants will first meet with the study coordinator to review the details of the study. Participants will be assigned a randomly generated Unique Research Subject Identifier (URSI) number, following standard protocol at MRN. To obtain an URSI, research staff first enter basic demographic variables including name, address, and contact information into an operational database (the URSI database). Each subject is then provided with a random unique number (URSI) to maximally protect the confidentiality of their data across all modalities and forms of data collection. In most cases, each subject is only assigned a single URSI number even if they participate in multiple studies. This URSI also serves as an operational link to the subject's identity. Therefore, it is important to note that the URSI database is for operational purposes only and can never be used for research purposes. To this end, only the PI (and their designated staff) who enrolled the subject into the research study will have access to any of the demographic information associated with the URSI while the study remains open at with the HRRC. Specifically, the database will not allow other research staff to access this basic demographic information associated with an URSI number to maintain subject confidentiality (i.e., it is password protected).

Participants will next provide informed consent (see attached consent form). They will then provide a urine sample in a private bathroom at for purposes of a toxicology screen. Additionally, female participants will complete a urine-based pregnancy screen (in the event of a positive result, the participant will not be allowed to continue with the study). Participants will next report to the UNM Psychiatry Department Pharmacy (in the building adjoined to MRN) for blood work. A licensed phlebotomist will acquire a small blood sample (approximately 12-15ml) to test for complete blood count (CBC) and a liver enzyme panel, including examining levels of gamma-glutamyl transferase (GGT), to ensure that participants are appropriate candidates for naltrexone

treatment. Blood samples will be labeled by URSI number and sent to TriCore laboratories. A TriCore representative will pick up the samples on the collection day.
When participants' blood/urine samples have been analyzed (estimated 2-3 days after the screening visit), results will be placed in the participant's research study folder at MRN for review by Dr. Arenella, along with results of the pregnancy screen. If the patient is deemed a candidate for naltrexone based on blood/urine screens (see exclusion criteria), they will be scheduled for their baseline (Week 0) appointment. At that time, participants will be asked to remain abstinent from alcohol for 72 hours prior to their appointment to permit an assessment of potential withdrawal. If the patient is not medically eligible for naltrexone based on blood/urine screens, the Dr. Arenella will call the participant to discuss the results, inform the participant of ineligibility, and provide a referral if indicated.

At the baseline (Week 0) visit participants will meet with the research assistant and Dr. Arenella to further determine whether naltrexone is medically appropriate. Inclusion/exclusion criteria related to psychiatric diagnoses/medication and substance use factors will initially be evaluated by a project assistant trained in conducting clinical interviews (Jessica Mickey). This interview will also include a CIWA (Clinical Institute Withdrawal Assessment) to assess potential withdrawal symptoms and a breathalyzer test to ensure a breath alcohol content of 0. A score of 8 or higher will be an exclusion criterion; if such an event occurs the participant will be immediately referred to the UNM Hospital emergency department and excused from further participation. If the participant appears to meet any other exclusion criteria, Ms. Mickey will consult with Dr. Arenella to verify that the participant should be excluded; Dr. Arenella will then follow up with the participant to discuss referral options.

If the participant fulfills study criteria based on this interview, the participant will proceed to meet with Dr. Arenella for a medical assessment immediately afterward. Assessments will be conducted in a CPR clinic room and will consist of a physical examination and brief medical history interview. The medical screen will also include assessment of whether the participant has a current primary care provider (PCP) to ensure that participants will have access to a physician to continue naltrexone following study completion, if indicated. If a participant does not have a PCP Dr. Arenella will refer the participant to the UNM primary care clinic and recommend they establish a PCP as soon as possible. For female participants, medical visits will include discussion of birth control during the study and participants will be required to complete a menstrual log, which will be reviewed by Dr. Arenella in subsequent medical visits.

If any medical or psychiatric contraindications are noted at the baseline visit (summarized under exclusion criteria) the participant will be informed that they do not meet full eligibility criteria, given a referral as appropriate, for the visit at $20/hour, and excused from further participation. If participants are considered good candidates for naltrexone based on the medical screening, Dr. Arenella will place an order with the UNM Psychiatry Department pharmacy for the prescription. Participants receiving naltrexone will also be provided with a wallet card to carry with them which, in case of any medical emergency, is intended to notify physicians, or emergency medical teams that the participant is taking naltrexone. This is important for the participant's safety given that naltrexone is an opoid antagonist, thus, administering opiate-based pain medications may be ineffective or dangerous and should be avoided (or implemented only with caution).

Next, the participant will continue the baseline appointment by completing a set of study questionnaires (see Table 1 below and Appendix II, Study Measures). Questionnaires will be administered by computer and will take approximately 1-2 hours to complete. Participants will be seated alone in one of the five private clinical assessment rooms at MRN. Computerized questionnaires will be delivered using a secure, web-based interface programmed by Talaria, which has been used in previous studies (see Appendix III for security information). Therefore, responses (identified only by URSI number) will be stored on a secure server maintained by Talaria Inc., and will not be accessible on local servers or computers after the participant has completed the measures.

Following completion of questionnaires the participant will meet with the study coordinator for detailed instruction on using the study cell phone and responding to prompts delivered by the cell phone. The participant's URSI number (which will have been randomly generated using the procedures outlined above) will be entered into the AGATE/SASED administrative interface, where computerized randomization will occur. This system is maintained by Talaria, but accessible via a web interface by the study research assistant using a secure password. At this time the participant will be asked to select a drinking goal: abstinence or moderation (moderation will be defined by the participant). Once the URSI number is entered, the computer randomization algorithm will block participants according to gender and drinking goal (abstinence or moderation), ensuring even distribution of these variables between conditions. The study coordinator will provide the participant with a study smartphone (Blackberry 8330 or equivalent) and instruct the participant in its use. This will include basic functions of the phone, how to to respond to SMS prompts, and guidelines for acceptable use of the phone during the study (described below). Participants will also provide a saliva sample for analysis of a genetic variant in the mu opioid receptor gene (OPRM1 A118G, rs1799971), which is demonstrated to influence response to naltrexone (Anton et al., 2008; Ray & Hutchison, 2007). Saliva will be collected in a plastic tube, labeled with the URSI number, and stored in a secure freezer in the neurogenetics lab at MRN. Samples will remain there until they are destroyed (10 years following study completion). Genetic analyses will be conducted in the neurogenetics lab at MRN. OPRM1 will be evaluated as a predictor of response to naltrexone in secondary analyses.

Finally, the participant will pick up his/her 4-week prescription for naltrexone at the UNM Psychiatry Department pharmacy. The pill container will be fitted with a MEMS monitoring system by the pharmacist, which will track the date/time of each time the container is opened. This data, which is stored in a microchip in the MEMS cap. The pillcap will not be linked to any personal identifier or URSI. The data will be downloaded when the participant returns for subsequent visits and labeled by URSI at that point. Data (timestamps) on pillcap openings will be linked to URSI in a password-protected spreadsheet kept on a password-protected computer at MRN. At the end of the baseline appointment participants will be encouraged to contact the study coordinator with any questions, concerns, or problems related to study participation. They will also be encouraged to contact Dr. Arenella directly with any questions about the study medication or side effects.

Following the baseline visit, the course of the medication trial will be 8 weeks. During this time participants will come back to MRN/UNM CPR for follow-up medical visits (at weeks 4 and 8). This timeframe is consistent with recommended clinical guidelines for monitoring patients on naltrexone (Anton, 2008). At the Week 4 visit, participants will first be interviewed by the research assistant, who will assess potential side effects using a standard side effects checklist. Results of the interview (i.e., any side effects reported) will be placed in the research folder for Dr. Arenella to review. Participants will also bring medication bottles for pill counts and uploading of MEMS pillcap data. They will report whether they have had any problems with their study phone or AGATE system.. Participants will report whether they have had any problems with their study phone. Any problems will be documented and addressed. Participants will also complete a brief subset of the study questionnaires again (e.g., assessing drinking and medication adherence in the last 4 weeks, see Table 1). Similar to the baseline visit, participants will be seated alone in a private assessment room at MRN and will complete measures using the web-based interface. Next, a follow-up blood sample will be obtained in the Psychiatry pharmacy using the same procedures and tests as described in the screening visit. Then the participant will meet with Dr. Arenella for a follow-up medical assessment. Dr. Arenella will review the contents of the participant's research study folder (e.g., side effect checklist) and interview the participant. If any medical contraindications to continuing naltrexone are evidenced, naltrexone treatment will be discontinued immediately. In these cases, Dr. Arenella will discuss with the participant an appropriate referral plan. To avoid threats to internal validity, Dr. Arenella will be blind to study condition (AGATE/SASED). For this reason, she will not discuss aspects of AGATE or barriers to adherence with participants; the interview will be focused on side effects and medical/safety issues. If no contraindications to continuing naltrexone are evidenced, participants will be provided with the next 4-week naltrexone prescription, which will be sent to the pharmacy. Again, pill vials will

be re-fitted with a MEMS monitoring system pillcap. Participants will not be allowed to take home their prescription on this day, pending results of the blood screen. When the results of the Week 4 blood screen are available, they will be placed in the study chart for review by Dr. Arenella. If no problems with the bloodwork are evidenced, the research assistant will contact the participant and ask that they pick up their prescription from the pharmacy. If any concerns are noted that suggest medication should be discontinued, Dr. Arenella will call the participant to inform them.

At week 8 the study physician will complete a visit identical to week 4 (i.e., it will include the same questionnaires, pill counts, blood draw, and medical visit). However, participants will be discharged at the end of the week 8 visit. At the medical assessment, Dr. Arenella will provide a discharge recommendation. Participants who show evidence of a good clinical outcome to naltrexone will be referred to their PCP. At the end of the 8 week visit the study coordinator will also conduct an exit interview to elicit qualitative information on the usability and acceptability of AGATE/SASED.

SMARTPHONE REMINDERS AND ASSESSMENTS

Overview of AGATE

Talaria has developed AGATE, an adaptive goal-directed adherence tracking and enhancement system for cell phones. To summarize, AGATE uses a combination of Short Message Service (SMS) text messages and internet access on a cellular handset to remind and assess medication adherence. On many handsets (e.g., Windows Mobile, iPhone, Blackberry, and others), SMS messages permit the inclusion of live hyperlinks that can launch web-based assessments, conducted via the internet. An administrator registers the participant in the database, using the email-to-SMS gateway address for the participant's phone. All major carriers in the US and abroad make is possible for their cellular subscribers to receive SMS messages via email by providing email addresses for this purpose. Thus, it is only necessary to know the participant's cell phone number and carrier. AGATE offers a simple and elegant yet powerful solution to the problem of medication adherence.

For a visual summary of the AGATE interface, as well as examples of questions assessing alcohol use and mediation adherence, please see Appendix IV for an excerpt from Talaria's technical report from the development of AGATE in the aforementioned Phase I SBIR study. Note that the Phase I study used HIV medication as the hypothetical target medication.

AGATE was designed so that the patient and provider agree upon (or the researcher sets) an adherence goal (e.g. a minimum of 90% of scheduled doses to be taken) over a certain period of time (e.g., 14 days). In the first stage, patients (or research participants) receive a reminder/assessment message for every scheduled dose. If at the end of this period the patient has achieved the goal, the patient progresses to Stage 2, in which the reminder/assessment frequency steps down (e.g., once every few days) for another period of time (e.g., 21 days). If at the end of this period the goal is maintained, the patient progresses to Stage 3, in which the reminder/assessment frequency steps down further (e.g., once per week). However, if the goal is *not* maintained in Stage 2 or 3, messaging frequency steps back up, and patients return to the preceding stage. In this context patients will be provided regular feedback on their performance and reminded of their goal. Participants will also complete daily assessments of drinking behavior (e.g., number of standard drinks per day, urge to drink) throughout the study using the AGATE system. Talaria will collect online metrics of number of messages ignored versus answered and latency to respond to messages to gauge the use/uptake of AGATE and SASED. For the current study, SMS reminders to take medication will be tailored based on the participant's level of adherence to that point. At the outset, participants will be reminded daily to take their medication. Upon achieving adherence for a period several days, reminders will be reduced to once every third day. Please see Appendix V for an example of the AGATE messaging parameters (frequency of reminders, etc).

Participants in the control (SASED) condition will not receive any medication reminders. However, all

participants (AGATE and SASED) will complete daily drinking measures (e.g., number of standard drinks per day, urge to drink) via the smartphone interface; these assessments will be sent each morning and will be identical across treatment condition.

Participants' Use of Study Cellphones.

Study cell phones will come activated with a 3-month pre-paid plan with Boost Mobile; these accounts will be maintained by Talaria. During this period participants will be assigned a phone number that will be linked to their URSI number in the Talaria database, but not to any identifying information. Cell phone plans will include unlimited minutes, texts and web access (this was the best option to avoid potential overage charges). Participants will also be allowed to keep their phone at the end of the study if they wish. However, to minimize the possibility of perceived coercion this will not be mentioned until after participants have enrolled in the study (i.e., after providing informed consent). Although participants will be allowed to use the phone for personal calls, texts, browsing, etc., they will be informed of certain limitations to cell phone use during the training provided at the baseline visit (see cell phone agreement form). For instance, participants will be instructed they cannot make international calls, cannot use the phone for illegal or unlawful purposes, and cannot attempt to sell, destroy or deface the phone during the course of the study. Over the course of the study, if the researchers deem that a participant is misusing the phone for these or other reasons, this may be grounds for removal from the study (as noted in the consent form). Once the participant completes the 8-week assessment they will be deemed discharged from the study and will no longer receive text messages from the study. They may continue to use the plan until it expires, at which point the phone number and plan will no longer be active. At that point participants can do as they wish with the phone (e.g., seek another service plan, give it away, etc.). If a participant discontinues the study before the 8-week appointment, they may be asked to return the phone at the discretion of the investigators. However, the investigators will not have any recourse for ensuring return of the phone. If the participant does not return the phone after a maximum of three phone call requests, we will not follow up further.

ii) Describe methods by which data will be managed - stored and destroyed.

Data Access/Sharing.

As described above, all data collected via cell phone and web-based questionnaires will be stored by Talaria on secure servers. Clinical data collected at MRN/UNM will be stored at MRN in the lab of Dr. Hutchison. All clinical data will be kept in research folders in a locked file cabinet, identified only by URSI number. For the purposes of data analyses at the end of the study, Dr. Hendershot will send a password-protected electronic data file to Dr. Stoner, containing coded clinical data (e.g., diagnostic data, side effects, etc.) which will be labeled only by URSI number. Data from the two sites will be merged by Dr. Stoner and sent back to Dr. Hendershot so both investigators can assist with data analyses.

Additionally, consistent with other studies in Dr. Hutchison's lab, we will seek participants' permission for data sharing in the event they participate in more than one study in our lab or at MRN. MRN has a standardized data sharing procedure based on URSI number). Participants will respond to a separate box on the consent form to indicate whether they agree to data sharing.

iii) Describe methods by which data will be analyzed and interpreted. Include the following, if applicable:

Data will be analyzed using traditional General Linear Model analyses, e.g., a mixed-groups ANOVA to evaluate main effects intervention condition (AGATE vs. SASED) pm adherence over the course of the study.

iv) Specific description of any experimental drug, device, or procedure. Any substance or instrument to be used in or on humans which has not received FDA approval for the proposed use requires an IND, IDE, or approval of the Radioactive Drug Research Committee.

N/A

v) Specific description of any use of biological samples. This is to include their origin, links to identifying information, storage, access, and future use.

Participants will provide a saliva sample for analysis of a genetic variant in the mu opioid receptor gene (OPRM1 A118G, rs1799971), which is demonstrated to influence response to naltrexone (Anton et al., 2008; Ray & Hutchison, 2007). Saliva will be collected in a plastic tube, labeled with the URSI number, and stored in a secure freezer in the neurogenetics lab at MRN. Samples will remain there until they are destroyed (10 years following study completion). Genetic analyses will be conducted in the neurogenetics lab at MRN. OPRM1 will be evaluated as a predictor of response to naltrexone in secondary analyses.

All participants will receive a statement summarizing The Genetic Information Nondiscrimination Act (GINA), which provides protection from risks of future discrimination based on genetic markers. All genetic material will be stored only with unique identifiers in the PI's laboratory. Participants will be informed in writing upon initial consent of the potential risks of genetic research. Consistent with our current protocols, the DNA sample will be kept for a period of 10 years at which time it will be destroyed. In the event that other single genetic markers (single nucleotide polymorphisms, or SNPs) are linked to response to naltrexone are identified, this will allow for analysis of those markers in the future. However, the planned analyses are presently limited to one genetic marker (OPRM1 A118G).

VI. Human Subjects:

i) Describe characteristics (inclusion criteria) of subject population, including precautions to be taken with vulnerable populations (e.g. children, prisoners, mentally ill/disabled person). Answer for each subject group, if different.

Participants and Recruitment. Participants will be heavy drinkers, (ages 21-55) with average weekly consumption ≥ 14 standard drinks for women and ≥ 21 standard drinks for men over the past 3 months (see below for full inclusion/exclusion criteria). Participants will be recruited primarily using posters/fliers, radio announcements, and newspaper advertisements, using procedures identical to a recently completed pharmacotherapy study for alcohol dependence conducted by Kent Hutchison, who is a co-Investigator in this proposal (HRRC# 07-228). In addition, we may recruit participants who have participated in other studies in the Hutchison lab, provided that they a) gave consent to be contacted about future studies, b) indicated at the time of participation that they were seeking treatment for alcohol problems or wanted to reduce their drinking (most of Dr. Hutchison's studies assess whether the participant is treatment-seeking), and c) meet all other eligibility criteria. Such participants will be identified using existing records and contacted by phone to assess interest in the study. We will not attempt to recruit participants who have taken medication as part of another study in Dr. Hutchison's lab. Participants who were screened for a recent study on olanzapine for alcohol dependence (HRRC# 07-228) and did not qualify based on reasons specific to olanzapine may be contacted and assessed for eligibility for treatment with naltrexone in this study.

Eligible participants must meet the following inclusion criteria:

1. Age 21-55
2. Report an average weekly consumption of ≥ 14 standard drinks for women and ≥ 21 standard drinks for men over the past 3 months (Anton et al., 2006).
3. Report at least two heavy drinking days (defined as 4+ drinks for women and 5+ drinks for men) in a consecutive 30-day period within the 90 days prior to baseline evaluation (Anton et al., 2006).
4. Participants must endorse a desire to reduce or stop drinking alcohol and willingness to try naltrexone.

5. Participants must receive medical clearance by the study physician to take naltrexone (based on blood panels and a medical evaluation).
6. Female participants of child-bearing age must report use of a birth control method deemed adequate by the study physician, must agree to continue this method over the course of the study, and must agree to contact the study physician if pregnancy is suspected during the study.
7.

Note: Because naltrexone is efficacious for reducing heavy drinking in non-dependent individuals (Kranzler et al., 2003), a diagnosis of alcohol dependence is not mandatory for inclusion in the study.

ii) Provide approximate number of subjects (both control and intervention groups).

Our goal is to randomize 105 participants. We anticipate this will leave us with 88 participants at study endpoint after accounting for attrition. Based on our previous experience with medication trials conducted at UNM/MRN, we anticipate conducting phone screenings for approximately 300 participants, of which approximately 140 will qualify to proceed to the in-person screening visit. Our liberal estimate is that 25% of these will be screened out for medical contraindications at the medical assessment before 105 are randomized.

iii) What characteristics (exclusion criteria) would exclude subjects, who are otherwise eligible, from this study? (Answer for each subject group, if different.)

Exclusion criteria will consist of the following:

1. Clinically significant physical illness (based on Dr. Arenella's medical evaluation).
2. Pregnancy or intended pregnancy among female participants.
3. Current (30-day) drug use other than alcohol, nicotine or cannabis (as verified by urine toxicology screen).
4. Any recent (90 day) use of opiate drugs or medications
5. Lifetime opioid dependence as assessed by a structured interview.
6. Currently taking any psychiatric medication other than antidepressants.
7. Lifetime diagnosis of a DSM-IV Axis I psychotic disorder.
8. CIWA score of 8 or higher, indicating clinically significant alcohol withdrawal, at baseline
9. Liver enzyme levels greater than 3x the upper limit of the normal range
10. Currently receiving another pharmacological or behavioral alcohol treatment, with the exception of Alcoholics Anonymous.
11. Currently court-mandated to seek treatment for alcohol problems, or otherwise required to document treatment for alcohol problems.

iv) Please see HRRC Recruitment Guidelines **Section 11.2** for more information about acceptable recruitment methods. Describe recruitment (source, initial contact method, etc.)

Described under i) above.

v) Describe Informed Consent procedures, including parental/guardian permission and minor assent for studies involving minors. Address any additional measures to be taken with any vulnerable populations. If you are requesting a waiver of informed consent, indicate request and justifications.

- Pre-enrollment: Participant completes phone screen.
- Screening visit: Research assistant provides participant with an overview of the study and provides the

consent form; participant provides informed consent by signing the consent form if they choose. Participant provides blood/urine samples.

- Week 0: Participant completes baseline medical evaluation. If all inclusion criteria and no exclusion criteria are fulfilled to that point, participant receives prescription and is issued a study smartphone.
- Weeks 4 and 8: Return visits for medical interview; blood draw; questionnaires.
- Weeks 0-8: Participant completes daily measures of drinking/adherence; those assigned to AGATE receive medication reminders.

For all recruitment and screening contacts emphasis will be placed on the voluntary nature of the study. Participants will be provided with as much time as they wish to read the consent form and decide upon participation. The ability for participants to withdraw without penalty anytime over the course of the study will be emphasized.

The payments are consistent with ongoing studies in Dr. Hutchison's lab, and are not large enough so as to be coercive. Although participants will be allowed to keep their cell phone following the study, we will minimize undue influence by informing them of this after enrollment, not during recruitment.
A trained research assistant will communicate with the participant over the phone and at the screening session. If there is any evidence that the participant does not understand certain information, efforts will be taken to carefully explain that information in more detail. If difficulties persist, the research assistant will notify the PI prior to allowing the participant to provide consent. The PI may decide against enrolling the participant if there is a persistent problem in successfully communicating information related to the study to a particular participant. The participant will sign the consent form, indicating they provide informed consent for participation. The form will be stored in a locked file cabinet separate from other information pertaining to the subject's participation. The participant will be given a copy of the consent form to take with them.

vi) Describe potential risks, including physical, psychological, social, economic, or legal, as well as those that might arise due to a breach of confidentiality.
(1) Identify their seriousness and likelihood.

The risks in this study involve procedures performed solely for the purposes of research. These risks include: psychological ratings, side-effects to the study medication, discomfort from interviewing, and unanticipated risks.

Psychological Ratings: Patients may find the battery of psychiatric and psychological tests tedious and boring, or intrusive. To aid the former, brief breaks will be given during assessment as needed. The latter concern is addressed through use of trained interviewers who have experience discussing sensitive material with clinical research subjects. It will be emphasized that participants can choose not to answer any question.

Medication: The most common side effects of naltrexone reported for the FDA-approved dosage used in this study are gastrointestinal discomfort, nausea, vomiting, headache, and fatigue (Anton, 2008) and a full list of side effects is listed in the consent form. Most side effects are mild and self-limiting and usually occur only during initial therapy. Naltrexone is relatively contraindicated in patients who have liver-enzyme levels that are four to five times above the upper limit of the normal range. Thus, blood tests of of hepatic enzymes should be obtained about a month after the initiation of treatment. Such tests can be repeated monthly during a 4-month course of treatment (Anton, 2008). Participants will be told not to take any opiate medication while on naltrexone. Female participants will be instructed to practice consistent contraceptive use (or abstinence) and not to attempt to become pregnant. They will also keep a menstrual diary that will be reviewed with the physician. Participants will be monitored closely throughout the study via side effects check-ups and two medical screens after starting medication.

Risks Pertaining to Loss of Confidentiality and Privacy: Confidentiality of participants is a priority for research staff and also is presumed and must be maintained unless the investigator obtains the express permission of the subject to do otherwise. Risks from breach of confidentiality include invasion of privacy, as well as social and economic risks. Economic risks include alterations in relationships with others that are to the disadvantage of the subject, and may involve embarrassment, loss of respect of others, labeling with negative consequences, or diminishing the subject's opportunities and status in relation to others. There are some risks related to the use of cell-phones to collect daily data on drinking and medication adherence. Participants might receive texts while in the company of others and be questioned as to the nature of the message. If a participant leaves his/her phone unattended, it is possible that someone else could view an incoming text message. We will take steps to instruct participants to always keep the phone close by and recommend that the participant sets a password on the phone.

Risks Pertaining to the Collection of Genetic Material: The collection of genetic material entails additional potential risks of confidentiality. However, the collection of genetic material has become commonplace, and entails only minimal risk to study participants. The Genetic Information Nondiscrimination Act (GINA), which recently came into effect at the Federal level, protects individuals from discrimination from empoyers or insurance companies as a function of genetic risk markers. All participants will receive a statement summarizing GINA. All genetic material will be stored only with unique identifiers in the PI’s laboratory. Participants will be informed in writing upon initial consent of the potential risks of genetic research. Consistent with our current protocols, the DNA sample will be kept for a period of 10 years at which time it will be destroyed. In the event that other single genetic markers (single nucleotide polymorphisms, or SNPs) are linked to response to naltrexone are identified, this will allow for analysis of those markers in the future. However, the planned analyses are presently limited to one genetic marker (OPRM1 A118G).

Unanticipated risks: Any study may involve risks that cannot be anticipated. Any identified risks will be reported immediately to UNMHSC HHRC for further consideration.

(2) Discuss alternative treatments where appropriate.

There are other treatments for alcohol dependence. These treatments include other medications (e.g., acamprosate, disulfiram), various psychosocial therapies, and Alcoholics Anonymous. Participants can also seek naltrexone through their own physician rather than through this study. Participants can also choose to seek no treatment. At the baseline screening, if participants express a desire for a different treatment (or do not qualify for the current study) they will be notified of other treatment options and encouraged to discuss these with their primary care physician.

(3) Identify circumstances for terminating the study.

The study can be terminated by the subject at any time for any reason. The subject will be discontinued from the study if he/she experiences a serious adverse event to the medication. The investigators may withdraw participants who do not follow the study protocol (e.g., participants who repeatedly fail to show for appointments or in the case of repeated/excessive non-response to cell phone prompts). Participants who voluntarily discontinue medication may be removed by the investigators, but will always be referred to other treatment resources. Given the nature of cellphone data collection in this study, we will include misuse of the phone (as described previously in this application) as a potential condition under which participants may be removed from the study by the investigators. Situations in which the entire study may be terminated early include the following: If the Principal Investigator or other governing official discovers serious concerns about subjects' safety; because study objectives have been obtained; or in the unlikely event that the Principal Investigator becomes unavailable

and no other additional investigators are able to succeed his role.

vii) Describe procedures for protecting against or minimizing likelihood of identified risks. Include any procedures that will be used to maintain confidentiality as applicable.

Protection Against Risk: Participants are informed of all procedures beforehand, and they must read informed consent forms and sign consent forms stating that they understand and agree to the procedures before beginning the study. The following protective measures will also be in place:
a) Breach of confidentiality is unlikely because all data are identified only by numeric code and are stored in locked filing cabinets in a designated locked PHI storage area. Identifying information will be collected only for the purpose of contacting participants for their follow-up assessments. The list linking the numerical ID code to the participant's identifying information will be maintained separate and secure from the computerized data files in a locked filing cabinet, and this information will be destroyed immediately after the clinical trial is complete. Dr. Stoner will oversee entry of all research instruments for the naltrexone trial (i.e., measures outside of AGATE and SASED) into the Talaria Test Builder (TTB). The TTB is Talaria's proprietary platform for web-based data collection.
b) Careful and consistent monitoring of potential side effects and/or adverse events throughout the study is in place. Furthermore, all subjects receive three blood tests (baseline, week 4, week 8), primarily to ensure that liver tests are within a normal range.
c) We will maintain an updated list of clinical services and local community practitioners for most psychiatric or substance disorder treatment services for participants, and we will provide appropriate referrals as necessary. Treatment providers in this study are qualified to dispense such advice and will do so when appropriate.
d) Normal patient handling procedures are followed to eliminate risks.

viii) Describe the expected benefits, including any possible direct benefit (such as alleviating a condition or providing a better understanding of a participant's condition.

The scientific benefits of the planned study are high, given the scientific importance of finding effective methods for maximizing adherence to pharmacological treatments for alcohol dependence. This study is expected to add to the knowledge base of information on adherence to naltrexone for alcohol problems. Also, it is expected that a proportion of participants will reduce their alcohol use during the current study and all participants will receive free access to an FDA-approved treatment for alcohol problems while enrolled in the study.
