# Supplementary figures and images for: Randomized Controlled Trial of a Mobile Phone Intervention for Improving Adherence to Naltrexone for Alcohol Use Disorders
Source: PLoS One. 2015 Apr 24;10(4):e0124613. doi: 10.1371/journal.pone.0124613 (PMC4409303; doi:10.1371/journal.pone.0124613)

# Alcohol and Side Effects Assessment

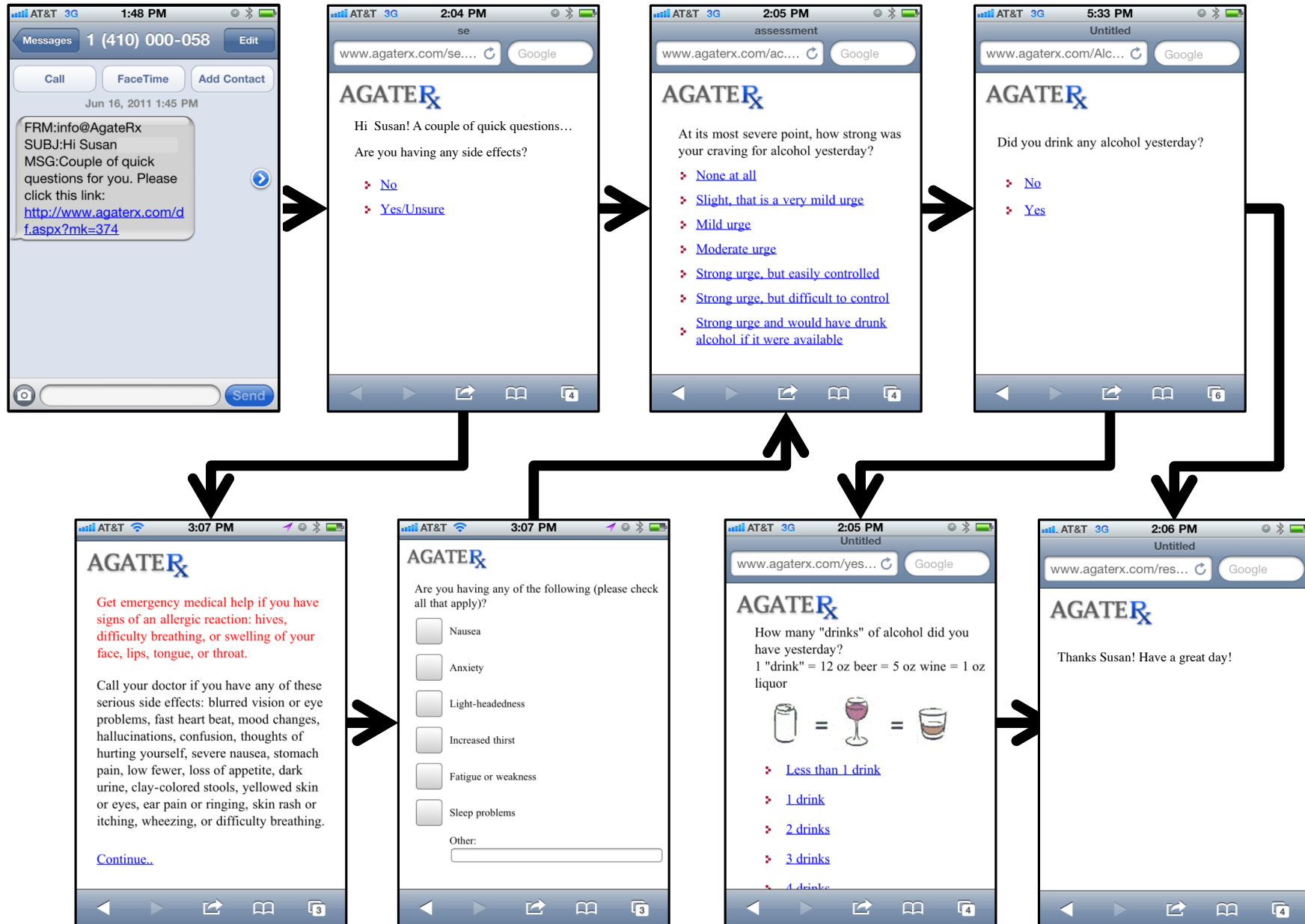

# Message and Adherence Assessment

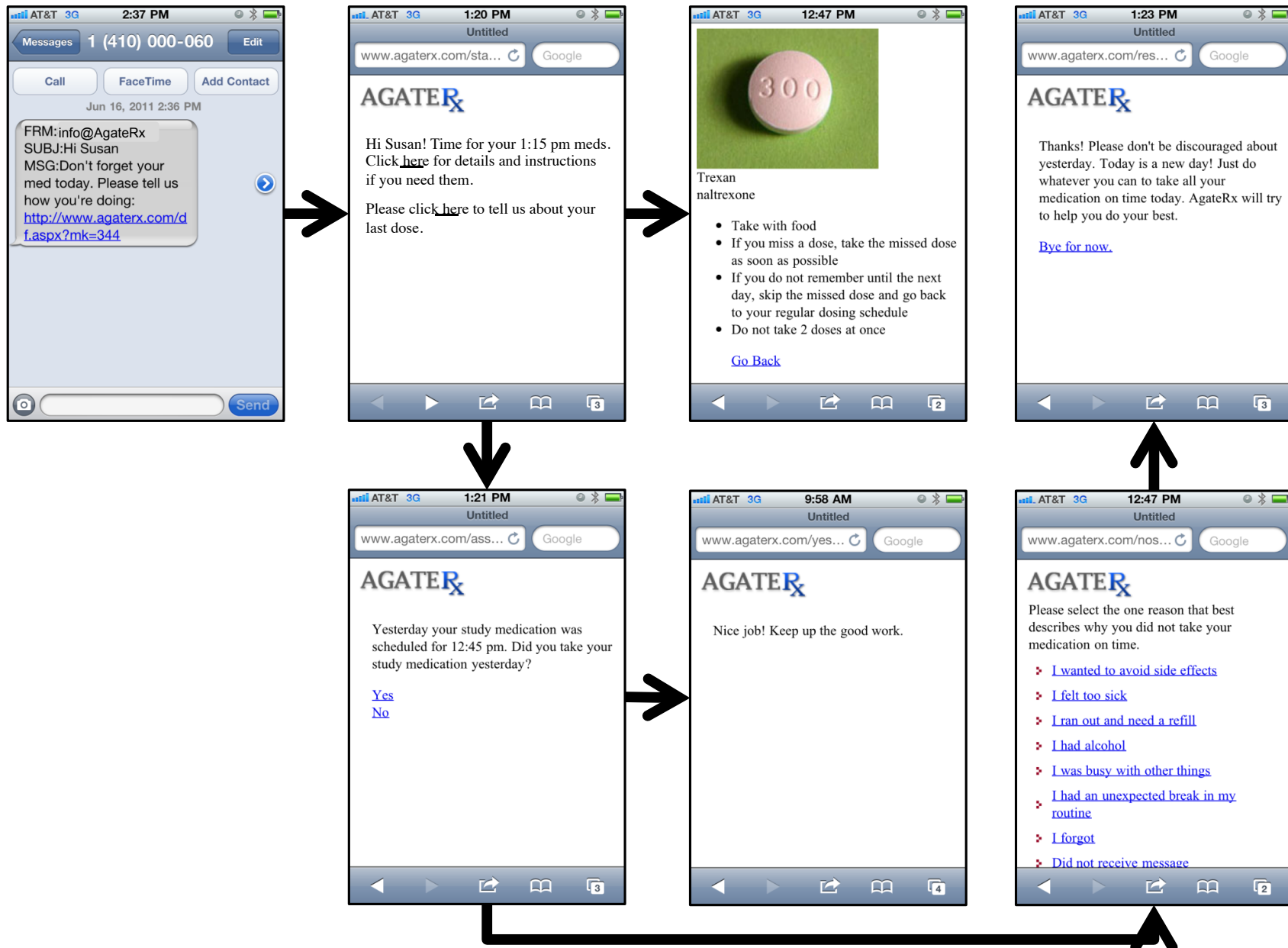

Supplement: S1 Screenshots — (PDF) [file pone.0124613.s004.pdf]
